# Functional connectivity and amplitude of low-frequency fluctuations changes in people with complete subacute and chronic spinal cord injury

**DOI:** 10.1038/s41598-022-25345-5

**Published:** 2022-12-03

**Authors:** Vanessa Vallesi, Johannes K. Richter, Nadine Hunkeler, Mihael Abramovic, Claus Hashagen, Ernst Christiaanse, Ganesh Shetty, Rajeev K. Verma, Markus Berger, Angela Frotzler, Heidrun Eisenlohr, Inge Eriks-Hoogland, Anke Scheel-Sailer, Lars Michels, Patrik O. Wyss

**Affiliations:** 1 Department of Radiology, Swiss Paraplegic Centre, Guido A. Zaech-Strasse 1, 6207 Nottwil, Switzerland; 2 Department of Diagnostic, Interventional, and Pediatric Radiology, University Hospital of Bern, Inselspital, University of Bern, Bern, Switzerland; 3 Division Imaging and Oncology, Image Sciences Institute, University Medical Center Utrecht, Utrecht, the Netherlands; 4 Digital Trial Intervention Platform, ETH Zurich, Zurich, Switzerland; 5 Clinical Trial Unit, Swiss Paraplegic Centre, Nottwil, Switzerland; 6 Outpatient Care Unit, Swiss Paraplegic Centre, Nottwil, Switzerland; 7 Department of Paraplegia, Rehabilitation and Quality Management, Swiss Paraplegic Centre, Nottwil, Switzerland; 8 Department of Neuroradiology, University Hospital Zurich, Zurich, Switzerland; 9 Neuroscience Center Zurich, University of Zurich and Swiss Federal Institute of Technology Zurich, Zurich, Switzerland

**Keywords:** Neuroscience, Trauma

## Abstract

After spinal cord injury (SCI), reorganization processes and changes in brain connectivity occur. Besides the sensorimotor cortex, the subcortical areas are strongly involved in motion and executive control. This exploratory study focusses on the cerebellum and vermis. Resting-state functional magnetic resonance imaging (fMRI) was performed. Between-group differences were computed using analysis of covariance and post-hoc tests for the seed-based connectivity measure with vermis and cerebellum as regions of interest. Twenty participants with complete SCI (five subacute SCI, 15 with chronic SCI) and 14 healthy controls (HC) were included. Functional connectivity (FC) was lower in all subjects with SCI compared with HC in vermis IX, right superior frontal gyrus (*p*_*FDR*_ = 0.008) and right lateral occipital cortex (*p*_*FDR*_ = 0.036). In addition, functional connectivity was lower in participants with chronic SCI compared with subacute SCI in bilateral cerebellar crus I, left precentral- and middle frontal gyrus (*p*_*FDR*_ = 0.001). Furthermore, higher amplitude of low-frequency fluctuations (ALFF) was found in the left thalamus in individuals with subacute SCI (*p*_*FDR*_ = 0.002). Reduced FC in SCI indicates adaptation with associated deficit in sensory and motor function. The increased ALFF in subacute SCI might reflect reorganization processes in the subacute phase.

## Introduction

In spinal cord injury (SCI), the afferent and efferent pathways in the spinal cord are damaged, resulting in long-lasting impairment of motor and sensory function^1^. Nonetheless, neuronal plasticity takes place in the spinal cord, which is a prerequisite for rehabilitation^2,3^. However, there is less known about the neuronal reorganization occurring in the brain and the extent to which this can be detected using neuroimaging methods.

In a resting-state fMRI study, altered local BOLD-signal correlation (regional homogeneity) in the sensorimotor regions, thalamus and cerebellum in acute SCI of maximal 30 days after onset was found^4^. These restructuring processes are also evident in functional connectivity (FC), namely the reduced covariance of the BOLD time series between primary and secondary somatosensory cortex in monkeys following SCI^5^.

Other connectivity measures such as the analysis of the amplitude of low-frequency fluctuations (ALFF), revealed alterations in complete SCI as reduced ALFF in the right lingual gyrus but increased ALFF in the right frontal gyrus^6^. This is also shown in the relative measurement of ALFF, namely fractional amplitude of low-frequency fluctuations (fALFF), where low fALFF was found in complete and incomplete SCI in superior medial frontal gyrus and higher fALFF in putamen and thalamus, which was negatively correlated with motor and sensory function^7^. The described changes might be different due to lesion characteristic, but also related to patient characteristics and therapeutic interventions^7^. Graph theory-based connectome analyses indicated decreased betweenness centrality in the left precentral gyrus, right caudal middle frontal gyrus and left transverse temporal gyrus in SCI^8^.

There are major differences in reorganization between humans with complete and incomplete disruption of the nerve signal from the corticospinal tract. In humans with SCI, lower FC has been reported in resting-state networks (salience, dorsal-attention, sensorimotor and default-mode networks) comparing in complete SCI to incomplete SCI^9^. Brain connectivity was lower in complete SCI than in incomplete SCI, and therefore the focus of this study was to investigate exclusively complete SCI.

In previous FC studies in SCI, the majority of the focus was on sensorimotor cortex^9–11^. Beside this area, however, the anterior and posterior portions of the cerebellum are also involved in motion and body representation^12,13^, making it relevant for SCI. Moreover, recent studies emphasize the role of the cerebellum in executive control ^14,15^. The cerebellar vermis, located between the two cerebellar hemispheres, has structural connections to motor areas. Using transneuronal tracers, a large number of neurons projecting from the motor cortex to the vermis were identified^16^. In addition to local proximity, strong relationship has been demonstrated between the vermis and cerebellum^16^. SCI induced in rats indicated cellular-level alterations in cerebellar circuits^17^.

Accordingly, in the case of deficits in somatosensory and motor activity, a series of reorganization processes is assumed to take place in the subcortical regions after a complete SCI, assuming that a longer duration of the SCI should result in a lower FC. In this study, we examined FC in the vermis and cerebellum in complete SCI. Our working hypothesis presumed that the functional connectivity is lower in complete SCI representing altered connectivity and is distinct between the subacute and chronic phase. The aim of this study is to clarify, first, brain connectivity and whether it is affected by the disruption of neural information from the lower limbs, and second, whether these impacts differ in the subacute compared to chronic phase after spinal cord injury.

## Results

### Demographics

A total of 36 subjects were recruited for the study at the outpatient clinics of our institution. All participants completed the MRI measurement and clinical assessments. Due to too high values in the Hospital Anxiety and Depression Scale (HADS) (≥ 7 score) indicating comorbidity of depression, two subjects had to be excluded in order to prevent the possible influence of depressive mood on resting state fMRI data. Thus, the final study sample consisted of 34 participants, 15 persons with chronic SCI (13 males, mean age = 53.5 ± 11.3 years, mean time since injury = 21.9 ± 13.6 years), 5 with subacute SCI (2 males, mean age = 39.4 ± 5.55 years, mean time since injury = 12.2 ± 4.8 weeks) and 14 HC (8 males, mean age = 41.2 ± 14.8 years) (see Table 1 for details of the SCI participants). The original target subsample of 15 participants with subacute SCI was not achieved due to recruitment difficulties. However, the total sample actually achieved is comparable to previous studies on SCI^9,18^. To determine whether there is a significant relationship between groups and sex, Fisher’s exact test was used. There was no statistically significant association between the groups and sex (two-sided *p* = 0.08). However, the Kruskal–Wallis test showed that there is a statistically significant difference between the groups and age (*X*^2^_(2)_ = 7.29, *p* = 0.03). Therefore, age was included as a covariate in all further analyses. The Kruskal–Wallis test examining the influence of motion showed that the three groups did not move on average differently during the fMRI scan. (*X*^2^_(2)_ = 0.177, *p* = 0.92).

**Table 1:** Demographics for spinal cord injury participants.

Subject	Sub-group	Sex	Age (in years)	Handedness	TSI (in years)	NLI	NP
1	cSCI	m	59	Right	34.89	Th4	Yes^a^
2	cSCI	m	57	Right	36.87	Th4	No
3	cSCI	m	49	Right	15.55	Th6	Yes^a^
4	cSCI	m	31	Left	4.91	Th6	No
5	cSCI	m	35	Right	12.79	Th6	Yes^b^
6	cSCI	m	65	Left	11.89	Th10	No
7	cSCI	m	57	Right	8.65	Th9	Yes^a^
8	cSCI	f	62	Right	22.91	Th2	No
9	cSCI	m	41	Right	11.39	Th10	No
10	cSCI	m	57	Right	50.26	Th3	No
11	cSCI	m	66	Right	35.16	Th3	No
12	cSCI	f	45	Right	37.19	Th5	No
13	cSCI	m	50	Right	18.97	Th4	No
14	cSCI	m	62	Right	19.28	Th6	No
15	cSCI	m	67	Right	8.50	Th11	No
16	sSCI	f	42	Right	0.35	Th10	No
17	sSCI	m	46	Right	0.24	Th6	No
18	sSCI	m	38	Left	0.31	Th11	No
19	sSCI	f	31	Right	0.14	Th3	No
20	sSCI	f	40	Left	0.16	Th4	Yes^a^

*cSCI* chronic SCI, *NLI* neurological level of injury, *NP* neuropathic pain, *sSCI* subacute SCI, *SCI* spinal cord injury, *TSI* time since injury.

^a^At neurological level, ^b^below neurological level.

### Connectivity analyses

**Figure 1:**
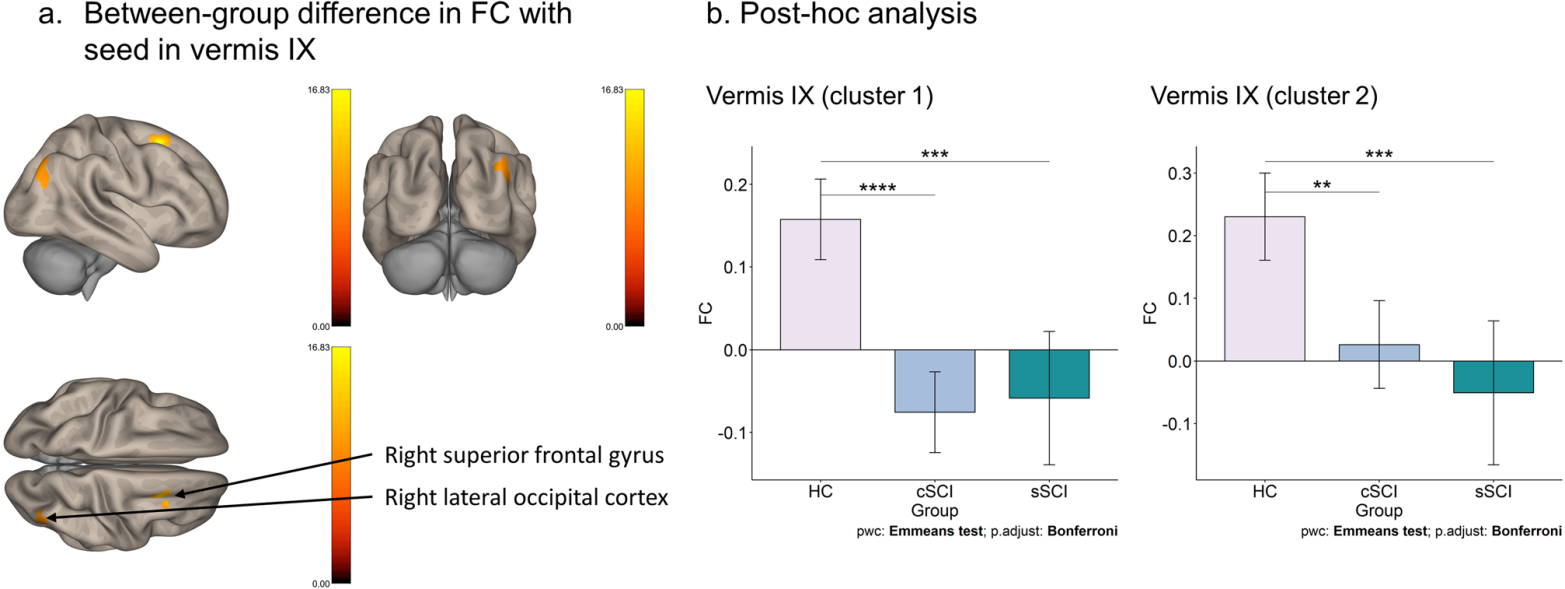
Functional connectivity group differences in the vermis. (**a**) The significant group differences of the functional connectivity (FC) in vermis IX between HC, cSCI and sSCI are shown (*p*_*FDR*_ < 0.05). (**b**) Estimated marginal mean (Emmean) FC is shown on the y-axis for the different groups on the x-axis for cluster 1 (right superior frontal gyrus) and cluster 2 (right lateral occipital cortex). All comparisons are Bonferroni corrected. Cluster 1: HC (Emmean = 0.16; Standard Error (SE) = 0.02); cSCI (Emmean = − 0.08; SE = 0.02); sSCI (Emmean = − 0.06; SE = 0.04). Cluster 2: HC (Emmean = 0.23; SE = 0.03); cSCI (Emmean = 0.03; SE = 0.03); sSCI (Emmean = − 0.05, SE = 0.06). **p < 0.01, ***p < 0.001, ****p < 0.0001.

The results of the seed-based FC analysis of covariance (ANCOVA) with age as covariate showed that there was a significant difference between HC vs. cSCI vs. sSCI with the seed vermis IX to the right superior frontal gyrus (*F*_(1,31)_ = 28.77, *p*_*FDR*_ = 0.008, η^2^ = 0.48, cluster size = 103 voxels) and to the right lateral occipital cortex (*F*_(1,31)_ = 25.07, *p*_*FDR*_ = 0.036, η^2^ = 0.45, cluster size = 73 voxels) as shown in Fig. 1. The Bonferroni post-hoc test revealed that cSCI have significant lower resting state FC compared to HC in both clusters (cluster 1: *t*_(1,31)_ = 6.56, *p*_*adj.*_ < 0.001, 95% CI [0.18, 0.29]) (cluster 2: *t*_(1,31)_ = 4.03, *p*_*adj.*_ = 0.001, 95% CI [0.15, 0.26]). There was also a significant difference between sSCI and HC (cluster 1: *t*_(1,31)_ = 4.81, *p*_*adj.*_ < 0.001, 95% CI [0.17, 0.26]) (cluster 2: *t*_(1,31)_ = 4.38, *p*_*adj.*_ < 0.001, 95% CI [0.19, 0.37]). The other vermis substructures were not significant regarding the FC in HC vs. cSCI vs. sSCI.

**Figure 2:**
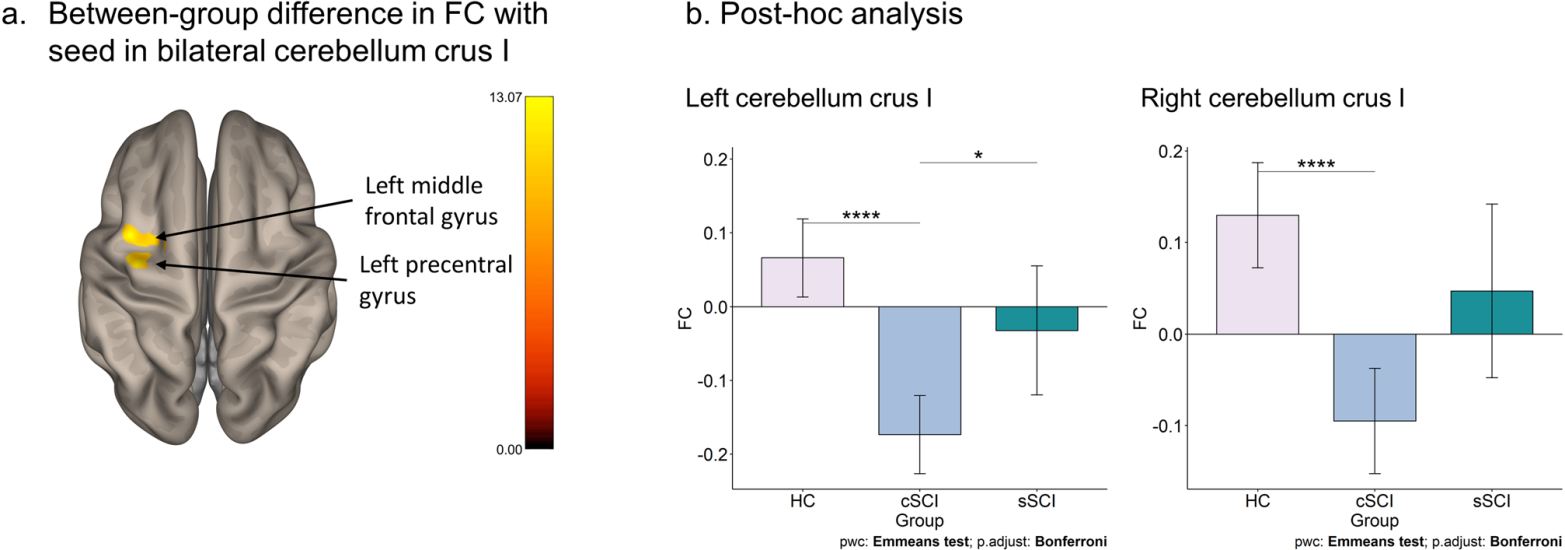
Functional connectivity group differences in the cerebellum. (**a**) The significant group differences in the bilateral cerebellum crus I in FC between HC, chronic SCI and subacute SCI are shown (*p*_*FDR*_ < 0.05). (**b**) Estimated marginal mean (Emmean) FC is shown on the y-axis for the groups on the x-axis. All comparisons are Bonferroni corrected. Left cerebellum crus I: HC (Emmean = 0.07; Standard Error (SE) = 0.03); cSCI (Emmean = − 0.17; SE = 0.03); sSCI (Emmean = − 0.03; SE = 0.04). Right cerebellum crus I: HC (Emmean = 0.13; SE = 0.03); cSCI (Emmean = − 0.10; SE = 0.03); sSCI (Emmean = 0.05; SE = 0.05). *p < 0.05, ****p < 0.0001.

Furthermore, there was a significant group means difference of FC in the cerebellum crus I left (*F*_(1,31)_ = 10.11, *p*_*FDR*_ = 0.001, η^2^ = 0.25, cluster size = 82 voxels and 65 voxels, resp.) and right (*F*_(1,31)_ = 7.08, *p*_*FDR*_ = 0.001, η^2^ = 0.19, cluster size = 82 voxels and 65 voxels, resp.) to the left precentral gyrus and the left middle frontal gyrus (see Fig. 2).

In the left cerebellum crus I, the post-hoc test showed that cSCI differed significantly from HC (*t*_(1,31)_ = 6.23, *p*_*adj.*_ < 0.001, 95% CI [0.18, 0.30]) and from sSCI (*t*_(1,31)_ = − 2.73, *p*_*adj.*_ = 0.032, 95% CI [− 0.10, − 0.18]). The cSCI group showed lower resting-state FC compared to HC and sSCI. In the right cerebellum crus I, FC in the cSCI was significantly different to HC (*t*_(1,31)_ = 5.38, *p*_*adj.*_ < 0.001, 95% CI [0.17, 0.28]), but not to sSCI (*t*_(1,31)_ = 1.56, *p*_*adj.*_ = 0.386). There was no significant difference between sSCI and HC (*t*_(1,31)_ = − 2.53, *p*_*adj.*_ = 0.051). No other cerebellar substructures differed within the three groups regarding FC.

The voxel-based morphological analysis did not reveal any differences in the grey matter volume (GMV) for any of the three ROIs with significant group FC differences (vermis IX: F_(2,31)_ = 0.094, p = 0.91; left cerebellum crus I: F_(2,31)_ = 0.346, p = 0.710; right cerebellum crus I: F_(2,31)_ = 0.295, p = 0.747).

**Figure 3:**
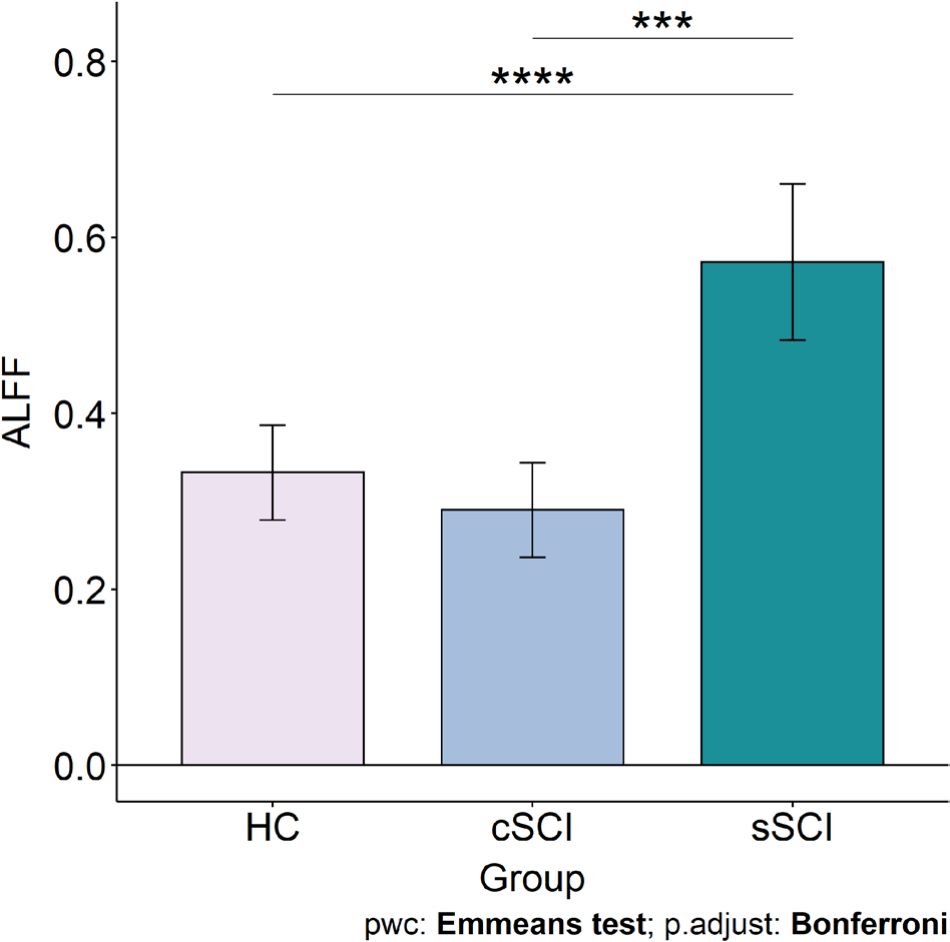
Group differences in the amplitude of low-frequency fluctuations (ALFF). Estimated marginal mean (Emmean) ALFF is shown on the y-axis for the groups on the x-axis. All comparisons are Bonferroni corrected. HC (Emmean = 0.33; Standard Error (SE) = 0.03); cSCI (Emmean = 0.29; SE = 0.03); sSCI (Emmean = 0.57; SE = 0.04). ***p < 0.001, ****p < 0.0001.

The results of the ANCOVA with the ALFF values revealed significant differences in the left thalamus (*F*_(1,31)_ = 9.04, *p*_*FDR*_ = 0.002, η^2^ = 0.23, cluster size = 41 voxels) between sSCI and HC (*t*_(1,31)_ = − 4.84, *p*_*adj.*_ < 0.001, 95% CI [− 0.16, − 0.32]) as well as sSCI and cSCI (*t*_(1,31)_ = − 5.35, *p*_*adj.*_ < 0.001, 95% CI [− 0.20, − 0.37]) (see Fig. 3).

There was no significant difference between the participant groups in the functional network analysis, neither for global efficiency nor for local efficiency.

## Discussion

In this study, a resting-state fMRI was conducted in participants with complete subacute and chronic SCI and HC at 3 T. Significant differences were found in several brain connectivity analyses between SCI and HC as well as between sSCI and cSCI.

The two SCI groups showed lowered FC compared to HC in vermis IX (uvula of vermis), superior frontal gyrus right and lateral occipital cortex. This specific vermis structure might be relevant for SCI since both SCI subgroups have lower FC than healthy controls. However, in the FC of bilateral cerebellum crus I, the left precentral gyrus and the left middle frontal gyrus, sSCI and HC were found to have a similar level of FC whereas cSCI showed a lower FC. These findings may indicate functional reorganization in SCI in the chronic phase. Differences in FC were found between cSCI and sSCI, which might be due to the state. In previous resting-state fMRI studies, reduced FC was also found in SCI, but so far only with the seed regions in the sensorimotor areas. Decreased FC was found in the primary motor and primary sensory areas in pre- and post-comparison of induced SCI in mice, with the post measurement during the chronic phase^19^. Lower FC in people with complete SCI was found in sensorimotor cortex, but also higher FC in the left postcentral gyrus and bilateral thalamus^11^. Furthermore, reduced FC was found in complete SCI in the right lingual gyrus and vermis III^6^. So far, the vermis IX subregion has been associated with spatial orientation^20^. According to our results, the substructures of the vermis and cerebellum seem to be relevant in relation to SCI, and future studies might analyze the subcortical area as single subdivisions.

Only sSCI showed higher ALFF in the left thalamus compared to cSCI and HC. This finding might reflect ongoing plasticity processes in sSCI, which might also be a factor contributing to the higher BOLD signal fluctuation. It has been shown that after upper limb amputation in humans, ALFF increases over time in thalamus, among others^21^. The ALFF results of this study are to some extent consistent with the pre-clinical results^22^, where non-human primates with induced SCI showed variations of fALFF in the left thalamus, left cerebellum, right lateral geniculate nucleus, right superior parietal lobule and posterior cingulate gyrus. It has been shown that 6 months after induced SCI in rats, i.e. the subacute phase, BOLD activity is increased in the thalamus, which has been linked to plasticity processes^23^. In a study with both complete and incomplete SCI in humans, where ALFF was examined in the early stage of the subacute phase (4–14 weeks), reduced BOLD fluctuation was found in the primary sensorimotor cortex and increased ALFF in the cerebellum and orbitofrontal cortex^10^. More in-depth research on ALFF following the time course of SCI is required as it shows effects on BOLD fluctuation especially in the early phase of SCI.

There was no difference between SCI and HC in terms of global and local efficiency in the network analysis, which is in line with previous results^24^. Similar to our findings, no network difference regarding global efficiency was found in subjects with complete SCI and HC, but they did show a significant difference in local efficiency^18^. However, the sample included patients with injury at the cervical level C4–C7 (tetraplegia), whereas in this study we only included paraplegia (i.e. injury at the thoracic level). It has also been shown that there are differences in reorganization between tetraplegia and paraplegia, as humans with tetraplegia showed lower brain activity in a positron emission tomography study^25^. Probably the lesion height and thus the proximity of the injury might lead to differences in neuronal networks efficiency. Future studies are required to investigate this association between injury level and functional networks.

Whether the lower FC in cSCI compared to sSCI and HC using the bilateral cerebellum crus I is due to the chronic phase remains open. Future studies are required to examine the time course of SCI with several measurement points from the acute to the chronic phase. Another issue is the definition of subacute and chronic phase in SCI as it is a continuous process between subacute and chronic phases. Therefore, in this study a clear cut-off point was chosen for the recruitment to ensure comparison of the two subgroups sSCI and cSCI. Consistent with the guidelines according to which the strongest recovery after SCI occurs in the first 3 months and lasts up to 18 months^26^, subjects with subacute SCI up to 7 months and subjects with chronic SCI from 2 years onwards were considered. Thus, the phase in between from the months 8 to 24 after SCI was not covered in order to achieve a more distinct separation. The sex ratio in our sample was unbalanced, with a larger proportion of men. However, this corresponds to the prevalence of SCI, where 68.3% are men^27^. Furthermore, the small sample in this study (especially that of the sSCI) is another limitation to generalization. To reduce the risk of alpha error (error type I), only adjusted p-values were considered, and the effect sizes were included. In addition, only people with complete paraplegic SCI were included in the sample to minimize strong variations within the group. This is relevant and therefore a strength of this study, as reorganization processes of incomplete SCI varies widely compared to complete SCI^9^.

Reduced FC was found in the substructures vermis IX and bilateral cerebellum crus I in complete SCI, especially in the chronic phase, which can be related to the deficit of sensory and motor activity of SCI. Increased ALFF was found only in sSCI, which provides evidence for plasticity processes, as it was not present in cSCI. Thus, this study demonstrates the relevance for future investigations in SCI involving the subcortical area. A detailed understanding of functional reorganization processes in cortical as well as subcortical regions will support therapy recommendations and rehabilitation in SCI.

## Methods

### Institutional review board approval

This cohort study was approved by the Institutional Review Board (local ethics committee northwest and central Switzerland (EKNZ), approval number: PB 2019-01624), and was conducted in accordance with the Declaration of Helsinki. To increase the quality of reporting of this observational study, STROBE guidelines were followed^28^. All participants signed an informed consent form before participating in the study.

### Participants

The recruitment took place from September 2019 to November 2021. The data were acquired on the same day. The inclusion age for all participants ranged from 18 to 80 years. The guidelines for clinical trials with SCI were followed for recruitment regarding the time since injury for SCI to differ between subacute and chronic state^26^. Therefore, the subsequent additional inclusion criteria were pursued for people with SCI: Less than 7 months (for subacute, sSCI) or more than 24 months (for chronic, cSCI) since the SCI, having a complete injury with American Spinal Injury Association Impairment Scale (AIS) A and a lesion level between Th1 to Th12 (paraplegic). The exclusion criteria for the overall sample were MRI contraindication, traumatic brain injury, and neurological or mental disorders assessed by a survey.

### Experimental design

#### Clinical assessments

All participants with SCI were assessed for severity and level of SCI by certified physicians with the International Standards for Neurological Classification of Spinal Cord Injury (ISNCSCI)^29^. Furthermore, the questionnaire HADS^30^ was conducted to control for comorbidity of depressive and/or anxiety symptoms (exclusion if score > 7), as depression has been reported to alter FC^31^. To assess neuropathic pain, which has been shown to increase FC in SCI^32^, the International Spinal Cord Injury Pain Classification (ISCIP) was carried out^33^.

#### Imaging acquisition

The neuroimaging data were acquired with a 3 T MRI unit (Philips Achieva, release: 5.4.1; Philips Healthcare, Best, the Netherlands) using a 32-channel head coil (Philips Healthcare). Participants were placed supine in the scanner. MR measurement sequences included a survey acquisition, anatomic acquisitions and resting state fMRI measurements with a total examination duration of 14 min.

The anatomic T1-weighted images were acquired with a repetition time (TR)/echo time (TE) of 8.12 ms/3.71 ms, flip angle of 8°, slice thickness of 1 mm, field of view (FoV) of 256 × 256 × 180 mm^3^, voxel size of 1 × 1 × 1 mm^3^ and bandwidth of 191 Hz. This resulted in a duration of 2 min 6 s. The functional T2-weighted echo-planar images were collected using a TR/TE of 2700 ms/26.7 ms, flip angle of 80°, voxel size of 3 × 3 × 3 mm^3^, bandwidth of 2116 Hz, FoV of 240 × 240 × 160 mm^3^ and 220 repetitions. The duration of this sequence took about 9 min 54 s. During the fMRI sequence, the participants were instructed to relax, think of nothing in particular, keep their eyes closed but stay awake. No music was played during this measurement. Immediately afterwards, all participants were asked whether they had adhered to it.

#### Data preprocessing

CONN toolbox, version 21b^34^, based on SPM, was used for analysis of functional MRI data. The following preprocessing steps were conducted: The first 10 images were excluded to allow the spin-system to reach steady state. The functional images were realigned according to the first image and co-registered to the anatomical images. Normalization was performed as well as segmentation with respect to Montreal Neurological Institute (MNI) space so that gray matter, white matter, and cerebrospinal fluid (CSF) tissue could be separated^35^. All spikes above five standard deviations of the global BOLD signal were removed. To reduce physiological influences, e.g. head movement, slow frequency fluctuations in the range of 0.008–0.09 Hz were filtered out in the BOLD signal. The motion correction included the three translational and three rotational regressors, and their derivatives. Finally, a Gaussian-smoothing kernel of 6 mm (isotropic) full-width-at-half-maximum was applied. The whole brain was parcellated into 274 regions according to the Brainnetome Atlas^36^.

### Functional connectivity and statistical analysis

Statistical analyses were done with CONN toolbox^34^ and R software^37^ using the package emmeans^38^. Cerebellum and vermis were used separately in their subdivisions as seed regions for seed-based connectivity maps. In the Brainnetome Atlas, these seed structures are segmented into fine-grained subdivisions, resulting in 10 substructures of the cerebellum in the left and right hemisphere respectively and eight vermis substructures. For the cerebellum, the left and right substructures were combined in the model as an average effect. Fisher-transformed bivariate correlations between the individual seeds and every voxel of the brain were calculated and implemented in an ANCOVA for assessing group differences with age as covariate. Only clusters with False Discovery Rate (FDR) correction and a significance threshold of *p*_*FDR*_ < 0.05 were considered thereby minimizing the alpha error (error type I). Post-hoc group comparisons were then calculated with a Bonferroni correction and a significance level of *p* < 0.05. Confidence intervals (CI) of 95% and effect sizes eta-squared (η^2^) were calculated for all significant results. Kruskal–Wallis tests examined the influence of age and average motion.

The graph theory-based measures of centrality (global efficiency) and locality (local efficiency) were used for the analysis of functional network organization^39^. This involves examining the nodes (regions of interest, ROIs) with their edges (functional connections) in the entire brain. Global efficiency is calculated using the average of the inverse distance between a node and all other nodes and local efficiency is computed using the inverse of the shortest path length between a node and each of its adjacent nodes^40^. Both network metrics were calculated using a cost-function threshold range of 0.1–0.5 with 0.01 step size, which means that only the 10–50% highest correlations are kept for comparison between the groups. This was done to minimize the false positive rate^40^.

In addition, but complementary to the FC, a comparison was made between the groups in terms of ALFF to investigate whether in the selected frequency range the BOLD signal strengths differed^41^.

To determine the influence of voxel composition, we calculated the GMV for the ROIs with significant results. Thereby, we subject-specific overlaid the results of the grey matter segmentation with the atlas.

## Data Availability

The data that support the findings of this study are available from the corresponding author upon reasonable request.
